# Development of TracMyAir Smartphone Application for Modeling Exposures to Ambient PM_2.5_ and Ozone

**DOI:** 10.3390/ijerph16183468

**Published:** 2019-09-18

**Authors:** Michael Breen, Catherine Seppanen, Vlad Isakov, Saravanan Arunachalam, Miyuki Breen, James Samet, Haiyan Tong

**Affiliations:** 1 Office of Research and Development, U.S. Environmental Protection Agency, Research Triangle Park, NC 27711, USA; isakov.vlad@epa.gov; 2 Institute for the Environment, University of North Carolina at Chapel Hill, Chapel Hill, NC 27517, USA; cseppan@email.unc.edu (C.S.); sarav@email.unc.edu (S.A.); 3 Office of Research and Development, ORISE/U.S. Environmental Protection Agency, Chapel Hill, NC 27514, USA; breen.miyuki@epa.gov; 4 Office of Research and Development, U.S. Environmental Protection Agency, Chapel Hill, NC 27514, USA; samet.james@epa.gov (J.S.); tong.haiyan@epa.gov (H.T.)

**Keywords:** mobile application, exposure model, inhaled dose, particulate matter, ozone

## Abstract

Air pollution epidemiology studies of ambient fine particulate matter (PM_2.5_) and ozone (O_3_) often use outdoor concentrations as exposure surrogates. Failure to account for the variability of the indoor infiltration of ambient PM_2.5_ and O_3_, and time indoors, can induce exposure errors. We developed an exposure model called TracMyAir, which is an iPhone application (“app”) that determines seven tiers of individual-level exposure metrics in real-time for ambient PM_2.5_ and O_3_ using outdoor concentrations, weather, home building characteristics, time-locations, and time-activities. We linked a mechanistic air exchange rate (AER) model, a mass-balance PM_2.5_ and O_3_ building infiltration model, and an inhaled ventilation model to determine outdoor concentrations (Tier 1), residential AER (Tier 2), infiltration factors (Tier 3), indoor concentrations (Tier 4), personal exposure factors (Tier 5), personal exposures (Tier 6), and inhaled doses (Tier 7). Using the application in central North Carolina, we demonstrated its ability to automatically obtain real-time input data from the nearest air monitors and weather stations, and predict the exposure metrics. A sensitivity analysis showed that the modeled exposure metrics can vary substantially with changes in seasonal indoor-outdoor temperature differences, daily home operating conditions (i.e., opening windows and operating air cleaners), and time spent outdoors. The capability of TracMyAir could help reduce uncertainty of ambient PM_2.5_ and O_3_ exposure metrics used in epidemiology studies.

## 1. Introduction

Epidemiological studies have found associations between exposure to ambient (i.e., outdoor-generated) fine particulate matter (PM_2.5_; particulate matter ≤2.5 µm in aerodynamic diameter) or ozone (O_3_) and indices of acute cardiopulmonary morbidity and mortality [1,2]. Most of these studies used outdoor PM_2.5_ or O_3_ concentrations as exposure surrogates due to the financial cost and participant burden from wearing personal air pollution measuring devices. However, these exposure surrogates do not account for building-to-building and temporal variations in indoor infiltration (i.e., attenuation) of ambient PM_2.5_ and O_3_, and variations in time spent in different indoor locations. Differences between exposure surrogates, such as outdoor concentrations, and true exposures, contribute to exposure measurement errors. Depending on the epidemiological study design, these errors can add bias or uncertainty to health effect estimates [3,4], which was highlighted in multiple reports by the National Research Council [5,6] and the National Academies of Sciences [7,8]. To address the recommendations in these reports, we developed an exposure model called TracMyAir, which is an iPhone application (“app”) that estimates near real-time individual-level exposures and inhaled doses to ambient PM_2.5_ and O_3_.

For TracMyAir, we extended a previously developed and evaluated exposure model called the Exposure Model for Individuals (EMI) [9,10,11,12]. The EMI predicts multiple tiers of individual-level exposure metrics for ambient PM_2.5_ using outdoor concentrations, questionnaires, weather, and time-location information. We used a mechanistic air exchange rate (AER) model, the mass-balance PM_2.5_ infiltration model, and a microenvironment-based exposure model to predict residential AER, infiltration factors, indoor concentrations, personal exposure factors, and personal exposures for ambient PM_2.5_. Using a cross-validation, individual predictions were previously compared to 591 daily measurements from 31 homes and participants in central North Carolina (NC). Median absolute differences were 39% (0.17 h^−1^) for AER, 18% (0.10) for infiltration factors, 20% (2.0 µg/m^3^) for indoor concentrations, 18% (0.10) for exposure factors, and 20% (1.8 µg/m^3^) for personal exposures [9,10].

The extended EMI for TracMyAir includes six additional capabilities. First, the TracMyAir exposure model includes both PM_2.5_ and O_3_, whereas EMI includes only PM_2.5_. Second, the residential AER model was extended to account for mechanical ventilation from window fans. Third, the residential infiltration model was extended to account for indoor PM_2.5_ removal from home air cleaners. Fourth, a ventilation model was added to predict inhaled dose from physical activity information. Fifth, an automated data retrieval capability was added that obtains real-time input data (i.e., ambient PM_2.5_ and O_3_ concentrations, temperature, and wind speed) to predict real-time exposure metrics for rapid, cost-effective exposure assessments. Finally, the exposure model was implemented as an iPhone application to facilitate and broaden the use of exposure metrics for epidemiological studies.

This manuscript demonstrates the capabilities of TracMyAir for use in future epidemiology studies. We will first describe the application’s model algorithms, inputs, and operating procedure; and then the method used to evaluate the application’s automated input functionality, and to perform a sensitivity analysis.

## 2. Materials and Methods

### 2.1. Overview of iPhone Application (TracMyAir)

**Figure 1:**
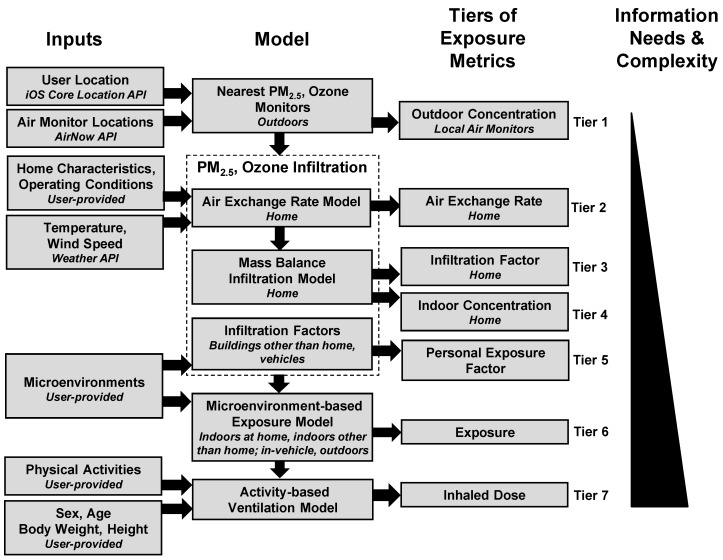
Conceptual model of TracMyAir to predict seven tiers of individual-level exposure metrics for ambient fine particulate matter (PM_2.5_) and O_3_. Tier 1 consists of measured outdoor concentrations, Tiers 2–4 are related to homes, and Tiers 5–7 are related to personal exposures and dose. Model input needs and complexity increase from Tier 1 to Tier 7.

We developed an iOS application for the iPhone smartphone (Apple Inc., Cupertino, CA, USA) to determine seven tiers of exposure metrics for ambient PM_2.5_ and O_3_ (Figure 1), which include measured outdoor concentrations at nearby monitors (Tier 1), three exposure metrics related to PM_2.5_ and O_3_ infiltration into homes, (Tier 2: AER; Tier 3: infiltration factors; Tier 4: indoor concentrations), two exposure metrics that account for time spent in different indoor and outdoor locations (Tier 5: personal exposure factors; Tier 6: exposures), and a metric that accounts for time spent at different intensity levels of physical activity (Tier 7: inhaled dose). The application determines individual-level exposure metrics from ambient air pollutant concentrations, weather, home building characteristics and operating conditions, time-location, and time-activity information. The application uses a residential AER model, infiltration model, a microenvironment-based exposure model, and an activity-based ventilation model. The application was written using Swift programming language (version 4.2.1; Apple Inc., Cupertino, CA, USA) and the XCode Integrated Development Environment (version 10.1; Apple Inc., Cupertino, CA, USA). Below, we describe the tiers of exposure metrics, and the method to operate the application.

Input data for the application are obtained for ambient PM_2.5_ and O_3_ concentrations, weather, home building characteristics and operating conditions, time-locations, and time-activities (Table 1). For the ambient air pollutant concentrations, and outdoor temperature and wind speed, the application automatically obtains these measurements from local air monitors and weather stations, respectively. The other inputs are provided by the user.

### 2.2. Tiers of Exposure Metrics

For the application, we developed seven tiers of 24-h average exposure metrics for PM_2.5_ and O_3_ (Figure 1). The tiers have increasing levels of complexity and information needs. Tier 1 is a measured exposure metric, whereas Tier 2–7 are modeled. The application calculates 24-h average exposure metrics for the previous four consecutive 24-h time periods (previous 96 h), which will allow future epidemiological studies to perform a lag analysis.

### 2.3. Measured Exposure Metric (Tier 1)

For Tier 1, TracMyAir uses the U.S. Environmental Protection Agency’s (EPA) AirNow application programming interface (API) to automatically obtain 1-h average PM_2.5_ and O_3_ concentrations from the closest official network air monitors based on the user’s location, and then calculates 24-h averages based on the past 96 h [13]. First, the application determines the user’s current geolocation (latitude, longitude) from the iOS Core Location API (Apple Inc., Cupertino, CA, USA). The Core Location can use all the geolocation methods available for iPhones (e.g., global positioning system (GPS), cell towers, and Wi-Fi), and automatically selects the most appropriate method to achieve the best level of accuracy available. For example, when GPS signal is unavailable (e.g., inside concrete and steel-framed buildings), the Core Location may use the geolocations of accessible Wi-Fi routers, or use triangulation based on signal strength of nearby cell towers.

Second, TracMyAir uses the AirNow API to determine the geolocations (latitude and longitude) of all PM_2.5_ and O_3_ monitors within a user-specified search radius (default = 60 km), and then calculates the distance to each monitor. The application then determines the closest monitor with a valid 24-h average. A 24-h average is considered valid if 1-h average measurements are available (i.e., value > 0) for at least 75 percent (i.e., 18 h or more) of the hours during the 24-h period, as defined in the EPA guidelines for the PM_2.5_ and O_3_ National Ambient Air Quality Standards [14,15]. If no valid monitors are found within the user-specified radius, TracMyAir displays a detailed error message and recommends the user to increase the radius and run the application’s test function for getting the air pollution monitoring data, as described below. Since some monitor sites do not measure both PM_2.5_ and O_3_, the closest monitor site for PM_2.5_ may be different than the one for O_3_. Additionally, if no valid monitors are found within a maximum search radius set to 75 km, TracMyAir displays a detailed message that the maximum search radius for the closest air pollution monitor has been exceeded and the estimated exposures may not be reliable from monitors beyond this distance.

### 2.4. Modeled Exposure Metrics (Tiers 2–7)

For Tier 2, residential AER are predicted from home building characteristics, home operating conditions, and weather (see Table 1) using a modeling approach that accounts for three types of airflows across building envelopes: (1) leakage from uncontrollable openings (e.g., cracks around windows and doors), (2) natural ventilation from open windows, and (3) mechanical ventilation from window fans.

For leakage and natural ventilation, we used the extended Lawrence Berkeley Laboratory model (LBLX), which is mechanistic in nature, accounting for the physical driving forces of the airflows (i.e., pressure differences across building envelopes from wind speed and indoor-outdoor temperature differences) [9,10]. The LBLX model was previously described and evaluated for homes in central NC and Detroit, Michigan [10,12]. Briefly, the leakage airflow is defined as

(1) $$Q_{\mathrm{leak}}=A_{\mathrm{leak}}\sqrt{k_{s}\left|T_{\mathrm{in}}-T_{\mathrm{out}}\right|+k_{w}U^{2}}$$

where *A*_leak_ is the effective air leakage area; *k*_s_ is the stack coefficient; *k*_w_ is the wind coefficient; *T*_in_ and *T*_out_ are the 24-h average indoor and outdoor temperatures, respectively; and *U* is the 24-h average wind speed (see Supplementary Material). The model has six user-provided inputs for home building characteristics (floor area, year built, number of floors, type of house, wind sheltering, and indoor temperature), and two automated inputs for weather (temperature and wind speed), as shown in Table 1.

For the outdoor temperature and wind speed, the application uses the National Weather Service API to automatically obtain 1-h average outdoor temperatures and wind speeds from the closest weather station based on the user’s location, and then calculates a 24-h average based on the previous 24 h [16]. This API automatically determines the nearby weather stations and ranks them from closest to furthest. The application then determines the closest monitor with a valid 24-h average. A 24-h average is considered valid if 1-h average measurements are available for at least 75 percent (18 or more) of the hours during the 24-h period. If the 24-h average is invalid, the next closest weather station is used. If no valid weather stations are found, the application displays an error message and recommends the user to run the test function for getting weather station data, as described below.

The LBLX model also accounts for natural ventilation airflow (*Q*_nat_) on days with open windows. The model has three user-provided inputs from window opening information (number of windows open, opening height, and opening duration), as shown in Table 1. The combined airflow for the leakage and natural ventilation airflows is defined as:

(2) $$Q_{\mathrm{LBLX}}=\sqrt{Q_{\mathrm{leak}}^{2}+Q_{\mathrm{nat}}^{2}}$$

The details are described in the supplementary material.

The application also accounts for mechanical ventilation airflow (*Q*_mech_) on days with residential window fans operating. The model has three user-provided inputs (operating duration, number of fans, and fan airflow), as shown in Table 1. The 24-h average airflow is defined as:

*Q*_mech_ = (*D*_fan_/24) *N*_fan_*Q*_fan_ (3)

where *D*_fan_ is the operating duration for the previous 24 h (h), *N*_fan_ is the number of window fans, and *Q*_fan_ is the airflow for a window fan (ft^3^/min). The default value for *Q*_fan_ is set to 600 ft^3^/min (1020 m^3^/h), which is the mid-range value for medium-size window fans (range: 300–900 ft^3^/min) [17].

The total airflow is defined as [18,19]

(4) $$Q_{\mathrm{total}}=\sqrt{Q_{\mathrm{LBLX}}^{2}+Q_{\mathrm{mech}}^{2}}$$

The AER is calculated as *Q*_total_ divided by the house volume *V*.

For Tier 3, residential infiltration factors (*F*_inf_home_) for PM_2.5_ and O_3_ were predicted with a steady-state mass balance infiltration model described by

*F*_inf_home_ = *P AER*/(*AER* + *k*_r_ + *k*_c_) (5)

where *P* is the penetration coefficient (dimensionless), *k*_r_ is the removal rate by indoor surfaces (h^−1^), and *k*_c_ is the removal rate of particles by air cleaners [20,21]. For PM_2.5_, *P* and *k*_r_ were previously estimated from homes in central NC (*P* = 0.84, *k*_r_ = 0.21 h^−1^) [9]. The parameter *k*_c_ is defined as

*k*_c_ = (*D*_c_/24) *CADR*/*V* (6)

where *D*_c_ is the air cleaner operating duration (h) for the previous 24 h, and *CADR* is the clean air delivery rate of the air cleaner (m^3^/h) [20,21]. The default value for *CADR* is set to 300 ft^3^/min (510 m^3^/h), which is the mid-range value for the top-rated portable air cleaners (range: 250–350 ft^3^/min) [22]. For O_3_, *P* and *k*_r_ were obtained from literature-reported values (*P* = 0.79, *k*_r_ = 2.80 h^−1^), and k_c_ was set to 0, since air cleaners are designed to improve air quality by removal of particulates, and no removal of indoor O_3_ is considered [23,24].

For Tier 4, residential indoor concentrations of ambient PM_2.5_ and O_3_ (*C*_in_home_) were predicted from measured outdoor concentrations from the nearest official monitor (*C*_out_) based on the steady-state equations [9,25]

*C*_in_home_ = *F*_inf_home_*C*_out_ (7)

For Tier 5, personal exposure factors of ambient PM_2.5_ and O_3_ were predicted as defined by

*F*_pex_ = *f*_in_home_*F*_inf_home_ + (*f*_in_work_ + *f*_in_school_ + *f*_in_other_)*F*_inf_other_bldg_ + *f*_in_vehicle_*F*_inf_vehicle_ + *f*_out_ (8)

where *f* are the user-provided inputs for the fraction of time spent across the previous 24 h in the six microenvironments (MEs: indoors at home, work, school, other; inside vehicles; outdoors). When the application is used to predict exposure metrics for the previous 4 days (96 h), the time spent in the six microenvironments is set to the same value for each 24 h interval. The *F*_inf_other_bldg_ and *F*_inf_vehicle_ are the infiltration factors for buildings other than homes and for vehicles, respectively. For PM_2.5_, we set *F*_inf_other_bldg_ to 0.64 based on the average of three literature-reported PM_2.5_ infiltration factors for offices, stores, and restaurants [26]. We set *F*_inf_vehicle_ to 0.44 based on literature-reported PM_2.5_ infiltration factor for cars [27]. For O_3_, we set *F*_inf_other_bldg_ to 0.12 based on the average of three reported O_3_ infiltration factors for offices, stores, and restaurants in central NC [28]. We set *F*_inf_vehicle_ to 0.23 based on reported O_3_ infiltration factor for cars [28].

For Tier 6, the total exposure to ambient PM_2.5_ and O_3_ is defined by [9,25]

*E* = *F*_pex_*C*_out_ (9)

The exposure from each ME is defined as

*E*_1_ = *f*_in_home_*F*_inf_home_*C*_out_ (10)

*E*_2_ = *f*_in_work_*F*_inf_other_bldg_*C*_out_ (11)

*E*_3_ = *f*_in_school_*F*_inf_other_bldg_*C*_out_ (12)

*E*_4_ = *f*_in_other_*F*_inf_other_bldg_*C*_out_ (13)

*E*_5_ = *f*_in_vehicle_*F*_inf_vehicle_*C*_out_ (14)

*E*_6_ = *f*_out_*C*_out_ (15)

where *E*_i_ is the exposure from each ME *i* where *i* = 1, 2, 3, 4, 5, or 6, corresponding to indoors at home, work, school, or other; inside vehicles; and outdoors, respectively. The percentage of exposure from ME *i* is defined by

*PE_i_* = 100 (*E*_i_/*E*) (16)

For Tier 7, the inhaled dose to ambient PM_2.5_ and O_3_ is defined as

*D*_ij_ = *E*_i_*MV*_j_*AT*_ij_/*BSA* (17)

where *D*_ij_ is the inhaled dose (µg/m^2^ body surface area) in ME *i* performing physical activity intensity level (PAL) *j*, where *j* = 1, 2, 3, and 4 correspond to sedentary (e.g., sleeping, sitting, or standing), light (e.g., walking <3 km/h, light cleaning, and cooking), moderate (e.g., walking >3 km/h or vacuuming), and vigorous (e.g., running), respectively [29,30]. The *MV*_j_ is the minute ventilation (L/min), *AT*_ij_ is the activity time spent (min) in ME *i* performing PAL *j*, and *BSA* is the body surface area (m^2^).

We determined age and sex-specific *MV* for each PAL *j* based on literature-reported normalized minute ventilations (NMV) (L/min/kg body weight; Tables S4–S11) [31]. The NMV were determined from oxygen consumption rates and basal metabolic rates based on data from the National Health and Nutrition Examination Survey and EPA’s Consolidated Human Activity Database. The NMV were reported for: (1) each PAL based on metabolic equivalent (METS) thresholds (sedentary: METS ≤ 1.5, light: 1.5 < METS ≤ 3.0, moderate: 3.0 < METS ≤ 6.0, and vigorous: METS > 6.0), (2) 14 separate age categories, and (3) both males and females. For the application, we used the reported median NMV for each PAL based on the user-provided age and sex. The *MV* is calculated as NMV multiplied by the user-provided body weight (kg).

The BSA is defined as

BSA = 0.007184 *BH*^0.725^*BW*^0.425^ (18)

where *BH* is body height (cm) and *BW* is body weight (kg) [32].

The total dose is calculated as

(19) $$D=\overset{6}{\underset{i=1}{\sum }}\overset{4}{\underset{j=1}{\sum }}D_{\mathrm{ij}}$$

The percentage of dose from each ME *i* and PAL *j* (*PD*_ij_) is defined by

*PD*_ij_ = 100 (*D*_ij_/*D*) (20)

### 2.5. Operation of TracMyAir

First, the application user selects either metric or English units, and then enters the user-provided inputs, which are automatically saved. Next, the user runs the exposure model and the application automatically determines and displays the seven tiers of exposure metrics for PM_2.5_ and O_3_ (Table 2; Figures S1 and S2). The application also outputs the geolocation and distance to the PM_2.5_ and O_3_ air monitors and weather station used by the exposure model (Figures S3 and S4). For subsequent analyses, such as for epidemiology studies, the application allows the user to save the model inputs and outputs in a text file and email the file to a user-specified address. It should be noted that the application also collects and outputs relative humidity from the weather station. The relative humidity is not used by the exposure model, but is often used for epidemiological analyses that examine short-term health effects from air pollution exposures.

The application allows the user to test the functionality and view the types of automated model input data: current user location, air pollution monitor data, and weather station data. For the user location, the application determines the phone’s current location, and displays the location on a map. For the air pollution monitoring data, the application determines the nearest PM_2.5_ and O_3_ monitors with valid 24-h averages, displays the monitor locations on a map, and shows the 1-h averages and 24-h averages. Similarly, for the weather station data, the application determines the nearest weather station with valid 24-h averages, displays the station location on a map, and shows the 1-h averages and the 24-h average.

The application has several user features. First, for the automatically-obtained model input data (air pollution and weather), the application allows the user to run the exposure model with user-provided values. With user-provided values, the exposure model can be run for specific scenarios, and tested without internet access. Second, the application also allows the user to modify parameter values for the residential infiltration model and ventilation model, and to set the distance to search for PM_2.5_ and O_3_ monitors. Thus, the application could support a broad range of studies that require different parameters and settings. Third, the application allows the user to set daily notifications that automatically prompt the user to run the application with new input data that changed in the past 24 h.

### 2.6. Evaluation of Automated Input Collection

We evaluated the ability of the application to automatically obtain real-time input data from the nearest PM_2.5_ monitor, O_3_ monitor, and weather station. The application was run at six different test locations across central NC. We used the application’s testing functions: “Get Air Pollution Monitor Data” and “Get Weather Station Data,” which display a map with a marker overlaid at the location of the nearest O_3_ monitor, PM_2.5_ monitor, and weather station. The application also calculates and saves the distances to the nearest air pollutant monitors and weather station. To determine the application’s accuracy, we used Google Earth (version 6.1.0.5001; Google, Mountain View, CA, USA) to determine the true distances from each of the six test locations to each of the four PM_2.5_ monitors, three O_3_ monitors, and two weather stations in central NC. Using a cursor, we selected the known locations of the user, PM_2.5_ and O_3_ monitors, and weather stations, and then the software automatically calculated the distances.

### 2.7. Sensitivity Analysis

To determine the effect on the exposure metrics to changes in six different model inputs (weather, window opening, window fan operation, home air cleaner operation, time-locations, and time-activities), we performed a sensitivity analysis. For the residential AER and infiltration models (Tier 2–3), we changed the indoor and outdoor temperatures and wind speeds (summer versus winter), windows (closed versus open), and air cleaners (none versus operating). For the exposure model (Tier 4), we changed the fraction of day spent outdoors. For the inhaled dose model (Tier 5), we changed the fraction of day spent at higher physical activity intensities.

The values for the sensitivity analysis are shown in Table 3 and Table 4. The default values for the various model inputs and the high and low values were set to reasonable values observed in previously reported field studies [9,10,33] and epidemiological studies [11,34] in central NC. The default values for *Q*_fan_ and *CADR* were set to the application default values.

## 3. Results

The TracMyAir inputs are provided in Table 1 for the automated inputs (outdoor PM_2.5_ and O_3_ concentrations, outdoor temperature, and wind speed) and user-provided inputs (home building characteristics and operating conditions, time-locations, time-activities, and demographics). For each input, the associated model and tier of exposure metric are provided. The application outputs are shown in Table 2 for the seven tiers of exposure metrics; as are statistics for the closest PM_2.5_ and O_3_ monitors and the nearest weather station; and the inhaled ventilations that were determined by the application.

For the evaluation of the application’s ability to automatically obtain real-time measurements from the nearest air pollution monitors and weather station, Table 5 shows the six different user locations and the distance to the four PM_2.5_ monitors, three O_3_ monitors, and two weather stations. For each user location, TracMyAir always correctly determined the closest air pollutant monitor and the closest weather station. Additionally, the distances to the closest monitors and weather station, automatically calculated by the application, matched those measured manually using Google Earth.

For the sensitivity analysis, we varied six different inputs and examined their effect on the different tiers of exposure metrics (Table 6). Details of the application input settings are provided in Table 3 and Table 4. We examined three types of inputs: weather, home operating conditions, and time-activities. For weather, we examined the sensitivity of the AER to changes in the indoor-outdoor temperature and the differences and wind speeds during the winter and summer. The AER was higher in the winter, as compared to the summer. This effect is due to a larger AER driving force from a higher indoor–outdoor temperature difference in the winter (15.2 °C) compared to the summer (0.5 °C). The wind speeds, and the resulting effect on the AER, were similar in the winter (4.8 km/h) and summer (5.0 km/h).

For home operating conditions, we examined the sensitivity of AER and *F*_inf_home_ to changes in opening windows, operating window fans, and using air cleaners. For windows, the AER, *F*_inf_home_ for PM_2.5_ and O_3_ were all higher on days with open windows compared to days with windows closed. This effect is due to the additional airflow from natural ventilation when windows are opened. For window fans, similar results are shown due to the additional airflow from mechanical ventilation. For air cleaners, the *F*_inf_home_ for PM_2.5_ was lower on days when air cleaners were used, and the *F*_inf_home_ for O_3_ was not affected. This effect is due to the additional removal of PM_2.5_ from indoor air by the air cleaners. Since air cleaners are designed to improve indoor air quality by the removal of particulates, no removal of O_3_ is considered.

For time-activities, we examined the sensitivity of daily exposure and dose to changes in daily time spent in different MEs and PALs, respectively. For ME, PM_2.5_, and O_3_, exposures are higher when a greater fraction of the day is spent outdoors. This effect is due to less time spent indoors where ambient levels are attenuated. Additionally, the percentage increase in exposure is greater for O_3_ than PM_2.5_. This effect is due to a higher percentage of O_3_ exposure occurring outdoors, since indoor attenuation of O_3_ is much higher than PM_2.5_. For PAL, PM_2.5_ and O_3_ doses are higher when a larger fraction of the day is spent doing higher intensity PAL with greater inhaled ventilations.

## 4. Discussion

Our goal was to develop a mobile application that can be used to predict multiple tiers of daily ambient PM_2.5_ and O_3_ exposure metrics for use in cohort health effect studies. Using TracMyAir, we can perform an individual-level exposure assessment for epidemiological studies that accounts for daily variations in ambient PM_2.5_ exposures and intake dose based on a mechanistic house-specific AER model linked to a mass-balance PM_2.5_ and O_3_ infiltration model, infiltration factors for nonresidential buildings and vehicles, and daily time-location and time-activity data from each participant. We previously demonstrated the ability to calibrate and evaluate the EMI for PM_2.5_ with extensive exposure data [9,10], and then apply EMI for the DEPS epidemiology study [11]. The impact of applying TracMyAir for epidemiological studies to improve health effect estimates will depend not only on the accuracy of the exposure assessment, but also other factors, such as the epidemiology study design [35]. The application calculates multiple tiers for exposure metrics with different levels of complexity and uncertainty, which can be used in epidemiological analysis to determine the benefit of more sophisticated exposure metrics.

TracMyAir can be applied in both short-term and long-term epidemiological studies, and in controlled human exposure studies. For short-term studies with daily health measurements across a few weeks, the application can provide daily 24-h average exposure assessments across multiple weeks, which will include the lag days that are often needed for the epidemiological analysis. Additionally, the application can provide daily time-specific notifications such that the daily exposures are time-matched with health measurements. In addition, the application could be used by the clinicians when collecting the health data during the participant’s visit to a human studies facility. For long-term exposure assessments, the application can provide daily reminders to users to enter any input data changes in their time-activity behavior (i.e., home operating characteristics, time-microenvironment, and time-activity), and to run the automated exposure calculation. Additionally, the application can account for participants that move to a new home during the study based on changing the home building characteristics, and the nearest weather station and air pollution monitors. For controlled human exposure studies, the application can be used to determine the participant’s air pollution exposure for the days prior to the controlled exposures at a laboratory.

There are several important features of TracMyAir. First, TracMyAir is based on the EMI, which was previously evaluated in three studies [9,10,11,12]. We evaluated Tiers 2–6 with 591 daily measurements of AER (Tier 2) and PM_2.5_ exposure metrics (Tiers 3–6) from 31 homes and participants across four seasons in central NC, which is the same geographical location and housing stock as an epidemiological study called PISCES conducted at EPA’s Human Studies Facility in Chapel Hill, NC. We are applying TracMyAir for PISCES, and upcoming EPA epidemiological and controlled clinical exposure studies in central NC.

Second, the application calculates 24-h average exposure metrics for the previous four consecutive 24-h periods. This allows epidemiological studies to perform a lag analysis of varying duration, which is a critical aspect of determining health effect estimates.

Third, the user can modify the model parameters. For example, this will allow a researcher to adjust the PM_2.5_ and O_3_ infiltration model parameters for each ME, which may be vary for different geographical locations and housing stock. Therefore, the application can be customized for specific studies, and used for scenario analysis and sensitivity analysis.

Fourth, the application can be run in a manual mode that uses only user-provided values for all model inputs. For example, for days with incorrect input data (e.g., housing characteristics), the exposure metrics can be retrospectively re-estimated by manually entering the correct input data. The automated input data, which was previously saved and emailed by the application, can be used to manually set the 24-h average outdoor PM_2.5_ and O_3_ concentrations, temperature, and wind speed.

Finally, the application can determine exposures in each ME, and inhaled doses for each PAL. For example, this allows a researcher to rank (e.g., highest to lowest) PM_2.5_ and O_3_ doses in each ME and for each PAL. This information could then be used to help design pollutant-specific mitigation strategies, such as modifying a building’s operation (e.g., open windows), time spent in different MEs, or performing different PALs (e.g., lower level) to reduce minute ventilation and inhaled dose.

We can compare TracMyAir to other mobile or website applications that are available for use in real-time exposure assessments in epidemiology studies. Most applications provide real-time outdoor air quality data for cities based on nearby monitor measurements and forecasted model predictions [36,37,38,39,40,41]. These applications are primarily designed to help the general public make informed decisions about their exposure risk during daily activities, and are not designed for scientific epidemiological studies. Unlike TracMyAir, these applications do not provide automated 24 h average outdoor concentrations time-matched to health outcome data, and do not account for the temporal variability and building-specific indoor attenuation of ambient PM_2.5_ and O_3_, time spent in different indoor and outdoor MEs, and PALs performed in each ME.

There are multiple and significant benefits of using TracMyAir for epidemiological studies. First, the application is simple to use, automatically obtains real-time outdoor ambient air pollution and weather input data, calculates real-time exposures, and runs on ubiquitous iPhones. Thus, it will broaden the range of applications for epidemiological studies. Second, the application determines exposures and inhaled doses in near real-time. Therefore, TracMyAir can be automatically time-matched with health data from epidemiological studies by running the application and sampling health effect data simultaneously. Third, the modeled exposure metrics account for the building-to-building and temporal variability of AER and the indoor attenuations of ambient PM_2.5_ and O_3_. Since people spend most of their time indoors, the variability of indoor attenuation can be a substantial source of variability in exposures between individuals, including studies across regions with small spatial variations in outdoor PM_2.5_ and O_3_ concentrations. Furthermore, when the outdoor PM_2.5_ and O_3_ concentrations are used as an exposure surrogate in epidemiological studies, the estimated health effect can be biased towards the null, since it is the product of the toxicity (i.e., true health effect) and the indoor attenuations of ambient PM_2.5_ and O_3_ [4]. Fourth, the application captures daily user-specific behavior (e.g., window opening, operating window fans, operating home air cleaners, time spent in microenvironments, and time performing different physical activities), and thereby accounts for the participant-to-participant and temporal variability of personal exposures and inhaled doses due to these behaviors. Finally, the application saves detailed information (e.g., location and distance to user) about the automated inputs (PM_2.5_ and O_3_ monitors, weather station), which are needed for reporting epidemiological findings.

One limitation of the application for long-term exposure assessments is the need for daily user-provided information. We designed the application to use input data from the past 24 h, instead of longer durations, to help reduce recall errors. Additionally, the time needed to enter daily data is minimal, since the application saves the input settings, and only daily changes need to be entered. There are three types of data (home operating characteristics, microenvironments, and physical activities) that may change daily, whereas the other two types of data (home characteristics and demographics) will typically not change. 

Another possible limitation of the application is the 24-h average exposure assessment. The temporal resolution of the exposure is limited by the 24-h average time-locations (i.e., duration in each ME for past 24 h). For some epidemiological studies, such as DEPS, this temporal resolution is sufficient. For studies that require higher temporal resolution, continuous GPS data linked with a microenvironment classification model, such as MicroTrac, can be used to determine continuous time-locations (i.e., time-of-day and duration in each ME) [33].

Another potential limitation is the use of measured PM_2.5_ and O_3_ concentrations from monitors potentially several kilometers from the user as input for the application. For PM_2.5_, we previously showed that for 31 homes in central NC, the modeled uncertainties for *E* and *C*_in_home_ were not substantially different using a central-site monitor or outdoor residential monitors for PM_2.5_ [9]. This is consistent with data from other cities in various U.S. regions that show PM_2.5_ mass concentrations are spatially homogeneous within each city, and that point and mobile sources have only limited influence [1]. This spatial homogeneity can be attributed to several factors, including slow settling velocity that results in long atmospheric lifetimes for PM_2.5_, and the significant fraction of PM_2.5_ that is from secondary origin [1]. Similar results have shown that O_3_ concentrations are also spatially homogeneous within each city [2]. O_3_ is a secondary pollutant, and therefore, is generally more regionally homogeneous than primary pollutants emitted from stationary or mobile point sources [2]. Furthermore, epidemiological studies often rely on ambient PM_2.5_ and O_3_ concentrations measured at a central monitoring site as exposure metrics [1,2]. For studies that require high spatial resolution, a fine-scale air quality model can be used [42]. We plan to investigate the feasibility of using an air quality model with high spatial resolution in a future application.

An additional limitation is the use of measured temperatures and wind speeds from nearby weather stations as inputs for the application. We previously showed that for 591 daily measurements from 31 homes and participants in central NC, the uncertainty of the residential PM_2.5_ infiltration model (*F*_inf_home_) was 18% (median absolute difference) using one central weather station for temperature and wind speed. For higher spatial resolution, a fine-scale weather model can be used [43]. We plan to investigate the feasibility of using a weather model with high spatial resolution in a future application.

## 5. Conclusions

This study demonstrates the ability of TracMyAir to predict multiple tiers of individual-level PM_2.5_ and O_3_ exposure metrics in near real-time. To improve exposure assessments, TracMyAir accounts for (1) the daily, house-specific infiltration of ambient PM_2.5_ and O_3_; (2) the daily, user-specific time spent outdoors, in-vehicles, and indoors at home and other buildings; and (3) the daily user-specific and microenvironment-specific time spent performing sedentary, light, moderate, and vigorous levels of physical activity. This capability can help provide more efficient and accurate exposure and inhaled dose estimates for epidemiological studies in support of improving health risk estimation.

## Figures and Tables

**Table 1:** TracMyAir inputs.

Categories and Model Inputs	Models	Tiers of Exposure Metrics	Method (Frequency)
Home characteristics Floor area, year built, number of floors, type of house (single family, multi-family), wind sheltering	Air exchange rate model	Tier 2	User-provided (one-time)
Home operating conditions			
Indoor temperature	Air exchange rate model	Tier 2	User-provided (daily)
Open windows Number of windows open, opening height, duration	Air exchange rate model	Tier 2	User-provided (daily)
Window fans Number of window fans, duration, airflow	Air exchange rate model	Tier 2	User-provided (daily)
PM_2.5_ air cleaners Number of air cleaners, duration, clean air delivery rate	Infiltration model	Tier 3	User-provided (daily)
Weather Temperature, wind speed	Air exchange rate model	Tier 2	Automated (daily)
Outdoor air pollution PM_2.5_, O_3_ concentrations	Infiltration, exposure models	Tiers 1, 4, 6	Automated (daily)
Microenvironments Time spent in 6 microenvironments	Exposure model	Tiers 5, 6	User-provided (daily)
Physical activity levels Time spent at 4 activity levels in 5 microenvironments	Activity-based ventilation model	Tier 7	User-provided (daily)
Demographics Sex, age, body weight, height	Activity-based ventilation model	Tier 7	User-provided (one-time)

**Table 2:** TracMyAir outputs.

Output	Description
Time period of exposure metrics	Start and end times for 24 h average exposure metrics
Weather Source (current location, user-provided), weather station ID, location, distance from user, temperature, wind speed	Closest weather station information
Ambient air pollution Source (current location, user-provided), PM_2.5_ and O_3_ monitor locations, distances from user, concentrations	Closest air monitor information, Tier 1 exposure metric
Home air exchange rate	Tier 2 exposure metric
Home infiltration factors for PM_2.5_ and O_3_	Tier 3 exposure metric
Home indoor concentrations for PM_2.5_ and O_3_	Tier 4 exposure metric
Personal exposure factors for PM_2.5_ and O_3_	Tier 5 exposure metric
Exposures for PM_2.5_ and O_3_ Total exposure, percentage from 6 microenvironments	Tier 6 exposure metric
Inhaled dose for PM_2.5_ and O_3_ Total dose, percentage from 6 microenvironments, 4 activity levels	Tier 7 exposure metric, microenvironment- and activity-specific doses
Ventilation rates Minute ventilation for 4 activity levels	Activity-specific minute ventilations

**Table 3:** Sensitivity analysis: inputs for outdoor air pollution, weather, home characteristics, and home operating conditions.

Model Inputs	Values [References]
Outdoor air pollution (24-h averages)	
PM_2.5_ concentration (µg/m^3^)	12.4 µg/m^3^ [11]
Ozone concentration (ppb)	26.0 ppb [34]
Weather (24-h averages)	
Temperature (°C)	18.4 °C (summer = 25.4 °C, winter = 7.3 °C [9,10]
Wind speed (km/h)	4.9 km/h (summer = 5.0 km/h, winter = 4.8 km/h) [9,10]
Home Characteristics	
Floor area (m^2^)	162 m^2^ [11]
Year built	1987 [11]
Number of floors	1 [11]
Type of house	Single family building [11]
Wind sheltering of house	Other buildings across street [11]
Home operating conditions (across 24 h)	
Average indoor temperature (°C)	23.8 °C (summer = 24.9 °C, winter = 22.5 °C) [9,10]
Open windows	
Number of open windows	0 (open windows = 4) [9,10,11]
Average opening height (cm)	0 (open windows = 15 cm) [9,10,11]
Duration windows open (h)	0 (open windows = 12 h) [9,10,11]
Window fans	
Number of window fans operating	0 (operating fan = 1) [34]
Duration fans operating (h)	0 (operating fan = 12 h) [34]
Airflow of window fans (ft^3^/min)	0 (operating fan = 600 ft^3^/min) [17]
Air cleaners	
Number of air cleaners operating	0 (operating air cleaner = 1) [34]
Duration air cleaners operating (h)	0 (operating air cleaner = 24 h) [34]
Clean air delivery rate (ft^3^/min)	0 (operating air cleaner = 300 ft^3^/min) [22]

**Table 4:** Sensitivity analysis: inputs for microenvironments, physical activities, and demographics.

Model Inputs	Values [References]
Microenvironments (duration across 24 h; hours:minutes) ^1^	Default (short time outdoors, long time outdoors) [9,10,11,33,34]
Outdoors	01:30 (00:15, 05:00)
Inside vehicles	01:00 (00:30, 00:30)
Indoors at work	07:45 (00:00, 00:00)
Indoors at other	00:30 (00:15, 00:15)
Indoors at home	13:15 (23:00, 18:15)
Physical activities (duration across 24 h; hours:minutes) ^1^	Default (low activity, high activity) [9,10,34]
Light intensity	
Outdoors	01:30 (00:30, 00:00)
Indoors at work	00:30 (00:15, 01:45)
Indoors at other	00:30 (00:00, 00:00)
Indoors at home	01:00 (00:15, 02:45)
Moderate intensity	
Outdoors	00:00 (00:00, 01:00)
Indoors at work	00:00
Indoors at other	00:00 (00:00, 00:30)
Indoors at home	00:00
Vigorous intensity	
Outdoors	00:00 (00:00, 00:30)
Indoors at work	00:00
Indoors at other	00:00
Indoors at home	00:00
Sedentary intensity	
Outdoors	00:00 (01:00, 00:00)
Indoors at work	07:15 (07:30, 06:00)
Indoors at other	00:00 (00:30, 00:00)
Indoors at home	12:15 (13:00, 10:30)
Inside vehicles	01:00
Demographics	
Sex	Male [11]
Age	64 [11]
Body weight (kg)	94 kg [11]
Height (cm)	175 cm [11]

^1^ Indoors at school (hours:minute) = 00:00.

**Table 5:** Evaluation of TracMyAir automated inputs for nearest outdoor air pollution monitors and weather stations.

User Test Location (City, County)	TracMyAir: Nearest PM_2.5_, O_3_ Monitors, Weather Station (Distance)	Google Earth: Measured Distance to PM_2.5_, O_3_ Monitors				Google Earth: Measured Distance to Weather Stations	
		Armory	Millbrook	RTP	RDU (No Ozone)	KRDU	KTDF
Hillsborough, Orange County	PM_2.5_: Armory (19 km) O_3_: Armory (19 km) Weather: KTDF (23 km)	19 km *	52 km	29 km	34 km	35 km	23 km *
Central Durham, Durham County	PM_2.5_: Armory (1 km) O_3_: Armory (1 km) Weather: KRDU (19 km)	1 km *	34 km	13 km	17 km	19 km *	33 km
South Durham, Durham County	PM_2.5_: RTP (1 km) O_3_: RTP (1 km) Weather: KRDU (8 km)	13 km	27 km	1 km *	5 km	8 km *	46 km
Raleigh, Wake County	PM_2.5_: Millbrook (6 km) O_3_: Millbrook (6 km) Weather: KRDU (17 km)	32 km	6 km *	24 km	19 km	17 km *	62 km
Morrisville, Wake County	PM_2.5_: RDU (6 km) O_3_: RTP (10 km) Weather: KRDU (7 km)	21 km	23 km	10 km **	6 km *	7 km *	55 km
Chapel Hill, Orange County	PM_2.5_: RTP (16 km) O_3_: RTP (16 km) Weather: KRDU (25 km)	17 km	44 km	16 km *	22 km	25 km *	43 km

* Indicates nearest air pollution monitors and weather stations for each user location; ** indicates second nearest air pollution monitor to obtain ozone measurements; RTP = Research Triangle Park air monitor site, RDU = Raleigh Durham Airport air monitor site, KRDU = Raleigh Durham Airport weather station, KTDF = Person County Airport weather station.

**Table 6:** Sensitivity analysis of TracMyAir outputs for six different input scenarios.

Model Inputs	Input Scenarios	Model Outputs	Effects on Exposure Metrics
Weather	Summer vs. winter	Summer: AER = 0.11 h^−1^, Winter: AER = 0.28 h^−1^	Higher AER in winter
Home windows	Closed vs. open windows	Closed: AER = 0.19 h^−1^, F_inf_home_ = 0.39, 0.05 (PM_2.5_, O_3_) Open: AER = 0.94 h^−1^, F_inf_home_ = 0.69, 0.20 (PM_2.5_, O_3_)	Higher AER, F_inf_home_ when opening windows
Home window fans	None vs. operating window fans	Closed: AER = 0.19 h^−1^, F_inf_home_ = 0.39, 0.05 (PM_2.5_, O_3_) Open: AER =1.30 h^−1^, F_inf_home_ = 0.72, 0.25 (PM_2.5_, O_3_)	Higher AER, F_inf_home_ when operating window fans
Home air cleaners	None vs. operating air cleaners	None: F_inf_home_ = 0.39, 0.05 (PM_2.5_, O_3_) Operating: F_inf_home_ = 0.09, 0.05 (PM_2.5_, O_3_)	Lower F_inf_home_ for PM_2.5_ when operating air cleaners
Microenvironments	Short vs. long time spent outdoor	Short time: Exposure = 5.0 µg/m^3^, 1.65 ppb (PM_2.5_, O_3_) Long time: Exposure = 6.5 µg/m^3^, 6.54 ppb (PM_2.5_, O_3_)	Higher exposure when longer time spent outdoors
Physical activity level	Low vs. high level	Low level: Dose = 35.7 µg/m^2^, 43.5 µg/m^2^ (PM_2.5_, O_3_) High level: Dose = 59.4 µg/m^2^, 115.5 µg/m^2^ (PM_2.5_, O_3_)	Higher dose when higher physical activity level

AER = air exchange rate; F_inf_home_ = home infiltration factor.

## Supplementary Materials

The following are available online at https://www.mdpi.com/1660-4601/16/18/3468/s1, Figure S1: Main screen for TracMyAir; Figure S2: TracMyAir output screen; Figure S3: TracMyAir map of the nearest PM_2.5_ and O_3_ monitors, and 24-h average concentrations; Figure S4: TracMyAir map of nearest weather station, and 24-h average temperature, and wind speed; Table S1: Stack coefficient *k*_s_ ((L/s)^2^/(cm^4^ K)); Table S2: Wind coefficient *k*_w_ ((L/s)^2^/(cm^4^ (m/s)^2^)); Table S3: Local sheltering; Table S4: Male sedentary ventilation rates (L/min/kg body weight); Table S5: Male light intensity ventilation rates (L/min/kg body weight); Table S6: Male moderate intensity ventilation rates (L/min/kg body weight); Table S7: Male vigorous intensity ventilation rates (L/min/kg body weight); Table S8: Female sedentary ventilation rates (L/min/kg body weight); Table S9: Female light intensity ventilation rates (L/min/kg body weight); Table S10: Female moderate intensity ventilation rates (L/min/kg body weight); Table S11: Female vigorous intensity ventilation rates (L/min/kg body weight).
