# Supplementary material for: Development of TracMyAir Smartphone Application for Modeling Exposures to Ambient PM2.5 and Ozone
Source: Int J Environ Res Public Health. 2019 Sep 18;16(18):3468. doi: 10.3390/ijerph16183468 (PMC6766031; doi:10.3390/ijerph16183468)
Supplement: Supplementary file 1 [file ijerph-16-03468-s001.pdf]

Supplementary Material

Air Exchange Rate (AER) Model

The AER has two parameters (k_s and k_w) and five inputs (A_{leak}, T_{in}, T_{out}, U, and V), where A_{leak} is the effective air leakage area; k_s is the stack coefficient; k_w is the wind coefficient; T_{in} and T_{out} are the indoor and outdoor temperatures, respectively; U is the wind speed; and V is the house volume. Parameters k_s and k_w were set to literature-reported values based on house-specific information on house height (number of stories) and local wind sheltering (Tables S1–S3). The user-provided number of stories and local wind sheltering can be determined from satellite and street-level images in Google Earth (version 7.1.7.2606; Google, Mountain View, CA, USA). The number of stories can be verified from online county and real estate databases of property records (Zillow, Seattle, WA, USA; Trulia, San Francisco, CA, USA). To determine V, we multiplied floor area by a ceiling height of 2.44 m (8 ft). The user-provided floor area can be obtained from the online county and real estate databases.

We estimate A_{leak} with a literature-reported leakage area model [1,2]. The A_{leak} is calculated as:

$$A_{\text{leak}} = \frac{NL}{NF} \tag{S1}$$

where NL is the normalized leakage and NF is the normalization factor. The NL is predicted from year of construction Y_{built} and floor area A_{floor} as described by:

$$\text{NL=exp}\ (\beta_0+\beta_1 Y_{\text{built}} +\beta_2 A_{\text{floor}}) \tag{S2}$$

where β_0, β_1, and β_2 are regression parameters. The user-provided Y_{built} and A_{floor} can be obtained from the online county and real estate databases. The NF is defined as:

$$NF = \frac{1000}{A_{floor}} (\frac{H}{2.5})^{0.3} \tag{S3}$$

where H is the building height. We set H to the number of stories multiplied by a story height of 2.5 m and adding a roof height of 0.5 m [1]. The parameters β_0, β_1, and β_2 were estimated by Chan et al. (2005) for conventional homes ($\beta_0 = 20.7$, $\beta_1 = -1.07 \times 10^{-2}$, and $\beta_2 = -2.20 \times 10^{-3}\text{m}^{-2}$).

For the LBLX model, the airflow from natural ventilation Q_{nat} can be calculated as:

$$Q_{\text{nat}} = \sqrt{Q^2_{\text{nat,wind}} + Q^2_{\text{nat,stack}}} \tag{S4}$$

where $Q_{nat,wind}$ and $Q_{nat,stack}$ are the airflows from the wind and stack effects, respectively. The $Q_{nat,wind}$ is defined as:

$$Q_{\text{nat,wind}} = C_v A_{\text{nat}} U \tag{S5}$$

where C_v is the effectiveness of the openings, and the A_{nat} is the area of the inlet openings. Using literature-reported values, we set C_v to 0.3 and A_{nat} to one-half of the total area of window openings [1]. When window opening area is not available, we set A_{nat} to one-half of the literature-reported value of 619 cm^2, which is the median daily total window opening area for homes in the same region of central NC as DEPS [1]. The $Q_{nat,stack}$ is defined as:

$$Q_{\text{nat,stack}} = C_{\text{D}} A_{\text{nat}} \sqrt{2g\Delta H_{\text{NPL}} |T_{\text{in}} - T_{\text{out}}| / max\{T_{\text{in}}, T_{\text{out}}\}} \tag{S6}$$

where C_D is the discharge coefficient for the openings, g is the gravitational acceleration, ΔH_{NPL} is the height from midpoint of lower window opening to the neutral pressure level (NPL) of the building, and $max\{T_{in}, T_{out}\}$ is the maximum value between T_{in} and T_{out}. Using literature-reported values, we set C_D to 0.65, the midpoint of lower window opening to 0.91 m, and NPL to one-half of H [1].

Table S1. Stack coefficient[1] k_s ((L/s)^2/(cm^4 K)).

	House Height (Stories)		
	**One**	**Two**	**Three**
Stack coefficient	0.000145	0.000290	0.000435

[1] ASHRAE Handbook-Fundamentals, 2009.

Table S2. Wind coefficient [1] k_w ((L/s)^2/(cm^4 (m/s)^2)).

Shelter Class	House Height (Stories)		
	**One**	**Two**	**Three**
1	0.000319	0.000420	0.000494
2	0.000246	0.000325	0.000382
3	0.000174	0.000231	0.000271
4	0.000104	0.000137	0.000161
5	0.000032	0.000042	0.000049

[1] ASHRAE Handbook-Fundamentals, 2009.

Table S3. Local sheltering [1].

Shelter Class	Description
1	No obstructions or local sheltering
2	Typical shelter for an isolated rural house
3	Typical shelter caused by other buildings across street from building under study
4	Typical shelter for urban buildings on larger lots where sheltering obstacles are more than one building height away
5	Typical shelter produced by buildings or other structures immediately adjacent (closer than one building height): e.g., neighboring houses on same side of street, trees, bushes, etc.

[1] ASHRAE Handbook-Fundamentals, 2009.

References

1. Breen MS, Breen M, Williams RW, Schultz BD. 2010. Predicting residential air exchange rates from questionnaires and meteorology: model evaluation in central North Carolina. Environ Sci Technol 44:9349-9356.
2. Chan WR, Nazaroff WW, Price PN, Sohn MD, Gadgil AJ. 2005. Analyzing a database of residential air leakage in the United States. Atmos Environ 39:3445-3455.
3. The 2009 ASHRAE Handbook-Fundamentals, American Society of Heating, Refrigerating, and Air Conditioning Engineers: Atlanta, GA, 2009.

Ventilation Rates for Inhaled Dose Model (L/min/kg body weight)

Table S4. Male sedentary.

Male Sedentary Intensity

VENTILATION RATES (L/MIN/KG BODY WEIGHT)	
1 year old	0.40
2 years old	0.34
3-5 years old	0.25
6-10 years old	0.16
11-15 years old	0.10
16-20 years old	0.08
21-30 years old	0.06
31-60 years old	0.07
61-80 years old	0.08
81 years and older	0.09

Table S5. Male light intensity.

Back Male Light Intensity

VENTILATION RATES (L/MIN/KG BODY WEIGHT)	
1 year old	1.01
2 years old	0.83
3-5 years old	0.63
6-10 years old	0.38
11-15 years old	0.24
16-20 years old	0.18
21-30 years old	0.15
31-70 years old	0.16
71-80 years old	0.17
81 years and older	0.18

Table S6. Male moderate intensity.

Male Moderate Intensity

1 year old	1.82
2 years old	1.54
3-5 years old	1.12
6-10 years old	0.71
11-15 years old	0.47
16-20 years old	0.38
21-40 years old	0.34
41-50 years old	0.35
51-60 years old	0.37
61-70 years old	0.34
71-80 years old	0.36
81 years and older	0.38

Table S7. Male vigorous intensity.

Male Vigorous Intensity	
1 year old	3.57
2 years old	2.87
3-5 years old	2.11
6-10 years old	1.38
11-15 years old	0.91
16-20 years old	0.69
21-30 years old	0.64
31-40 years old	0.62
41-50 years old	0.63
51-60 years old	0.64
61-70 years old	0.61
71-80 years old	0.63
81 years and older	0.70

Table S8. Female sedentary.

Female Sedentary Intensity	
VENTILATION RATES (L/MIN/KG BODY WEIGHT)	
1 year old	0.42
2 years old	0.35
3-5 years old	0.25
6-10 years old	0.16
11-15 years old	0.09
16-20 years old	0.07
21-50 years old	0.06
51-80 years old	0.07
81 years and older	0.08

Table S9. Female light intensity.

Back Female Light Intensity	
VENTILATION RATES (L/MIN/KG BODY WEIGHT)	
1 year old	1.04
2 years old	0.89
3-5 years old	0.60
6-10 years old	0.38
11-15 years old	0.22
16-20 years old	0.17
21-40 years old	0.15
41-60 years old	0.16
61-70 years old	0.14
71 years and older	0.16

Table S10. Female moderate intensity.

Female Moderate Intensity	
1 year old	1.87
2 years old	1.58
3-5 years old	1.11
6-10 years old	0.71
11-15 years old	0.43
16-20 years old	0.35
21-30 years old	0.32
31-40 years old	0.30
41-50 years old	0.32
51-60 years old	0.33
61-70 years old	0.28
71-80 years old	0.30
81 years and older	0.33

Table S11. Female vigorous intensity.

Female Vigorous Intensity	
1 year old	3.24
2 years old	2.81
3-5 years old	1.90
6-10 years old	1.33
11-15 years old	0.85
16-20 years old	0.69
21-30 years old	0.63
31-40 years old	0.59
41-50 years old	0.64
51-60 years old	0.61
61-70 years old	0.53
71-80 years old	0.58
81 years and older	0.63

TracMyAir Screenshots

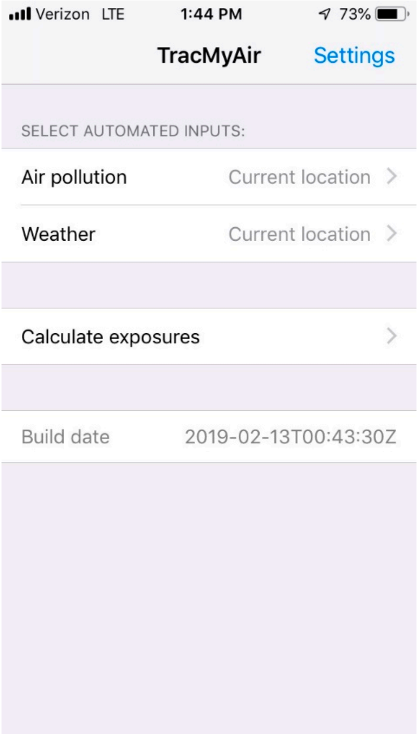

Figure S1. Main screen for TracMyAir.

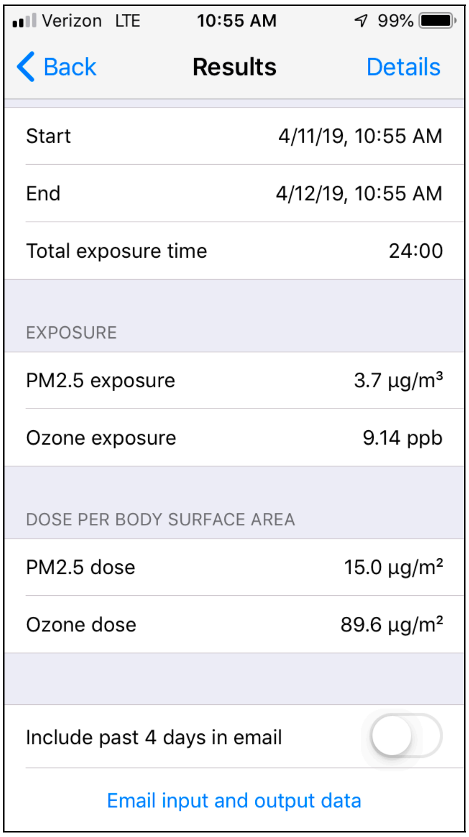

Figure S2. TracMyAir output screen.

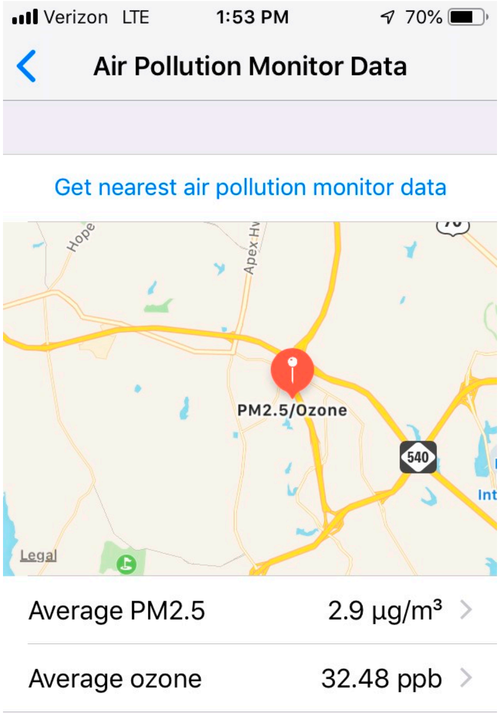

Figure S3. TracMyAir map of the nearest $PM_{2.5}$ and O_3 stations, 24-h average temperature, and wind speed.

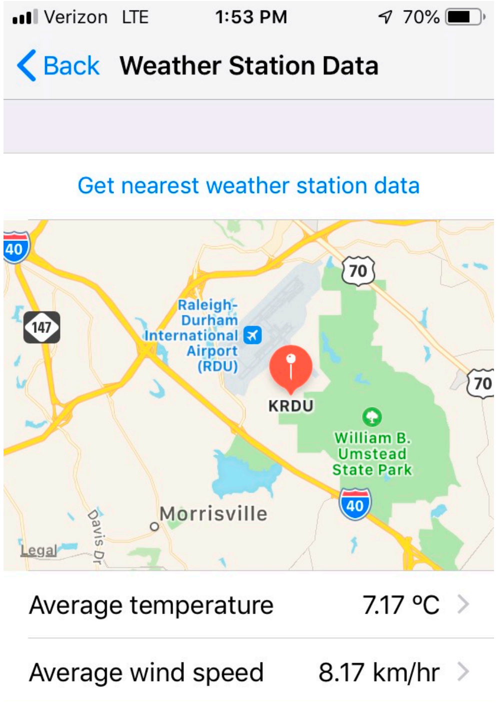

Figure S4. TracMyAir map of nearest weather monitors, and 24-h average concentrations.
